# FOXO1, TGF-β Regulation and Wound Healing

**DOI:** 10.3390/ijms150916257

**Published:** 2014-09-15

**Authors:** Alhassan Hameedaldeen, Jian Liu, Angelika Batres, Gabrielle S. Graves, Dana T. Graves

**Affiliations:** 1 School of Dental Medicine, University of Pennsylvania, Philadelphia, PA 19104, USA; E-Mails: ahame@dental.upenn.edu (A.H.); abatresnavarro@gmail.com (A.B.); 2 Department of Stomatology, the 4th Hospital of Hebei Medical University, Shijiazhuang 050011, China; E-Mail: zjh888fff@163.com; 3 Department of Medicine, Medical College of Wisconsin, Milwaukee, WI 53226, USA; E-Mail: ggraves@mcw.edu

**Keywords:** cell death, epithelial, FOXO, transforming growth factor-beta, migration, proliferation, repair, ROS, skin, wound

## Abstract

Re-epithelialization is a complex process that involves migration and proliferation of keratinocytes, in addition to the production of cytokines and growth factors that affect other cells. The induction of transcription factors during these processes is crucial for successful wound healing. The transcription factor forkhead boxO-1 (FOXO1) has recently been found to be an important regulator of wound healing. In particular, FOXO1 has significant effects through regulation of transforming growth factor-beta (TGF-β) expression and protecting keratinocytes from oxidative stress. In the absence of FOXO1, there is increased oxidative damage, reduced TGF-β1 expression, reduced migration and proliferation of keratinocytes and increased keratinocytes apoptosis leading to impaired re-epithelialization of wounds.

## 1. Introduction

Skin acts as a barrier, protecting the host from external forces and pathogenic organisms. Wounds allow foreign materials and organisms entry into the body. Therefore, wound healing is an important adaptive response against infection and is needed for the maintenance of homeostasis [1]. The healing process begins immediately after injury and occurs in three phases: inflammation and migration, proliferation, and remodeling and maturation [2]. Inflammation and migration involve degranulation of platelets and recruitment of neutrophils to the site of injury followed by recruitment of other leukocytes such as macrophages, which produce growth factors and cytokines [1,2]. Depending on the size of the wound and specific conditions, proliferation occurs during days 2–7 of wound healing and results in the formation of extracellular matrix and re-epithelialization [3,4]. Remodeling and maturation are the final stages in which the wound stabilizes and the proliferation of cells decrease [1,4]. Apoptosis, programmed cell death, is crucial to the cessation of tissue repair [5,6].

## 2. Inflammation

Neutrophils arrive at injured sites and phagocytize bacteria and debris, in addition to producing cytokines that aid in the recruitment of cells needed for revascularization and tissue restoration [6,7]. Neutrophils synthesize cytokines, such as IL-1, IL-6 and transforming growth factor (TGF)-α. These cytokines are important in regulating humoral and innate immunity, including the stimulation of macrophages and lymphocytes [6,7]. Macrophages, which arrive later, produce cytokines, growth factors, and angiogenic factors that regulate fibroblast proliferation and angiogenesis [8]. Macrophages produce IL-1α, IL-1β, IL-6, IL-12 and TNF-α, which are important in enhancing inflammation, and stimulate chemokines that induce the recruitment of leukocytes and stem cells [8]. Growth factors such as fibroblast growth factor (FGF), TGF-β, TGF-α and platelet derived growth factor (PDGF) stimulate migration and chemotaxis of keratinocytes and endothelial cells [8]. Chemokine ligands CXCL11, CXCL10 and CXCL4 and its receptor CXCR3 are essential for dermal maturation. CX3CL1 plays a role in attracting bone marrow-derived monocytes [8]. Inflammation later subsides in part due to apoptosis of leukocytes and the effect of mesenchymal stem cells that enhance the formation of anti-inflammatory regulatory T cells, which, in turn, reduce the proliferation and activation of natural killer cells, dendritic cells, and macrophages [6,8].

During the inflammatory phase, bone marrow releases multi-potent progenitor/stem cells into the blood stream [3]. Stem cells contribute to the proliferation and migration of epithelial cells and angiogenesis by releasing vascular endothelial growth factor (VEGF), insulin-like growth factor (IGF)-1, epidermal growth factor (EGF), keratinocyte growth factor, angiopoietin-1, stromal derived factor-1, CCL3, CCL4 and erythropoietin [3,8]. Epithelial stem cells, which are released from the hair follicles and sweat glands, contribute to the re-epithelialization process in wound repair [8,9]. In addition, basal keratinocytes at the wound edges are released from the basement membrane by the action of matrix metalloproteinases and migrate from the wound edge under the fibrin clot to participate in re-epithelialization [2,8].

Epithelial stem cells in the skin are divided into three subgroups: inter-follicular epidermal stem cells, hair follicle stem cells and stem cells of other ectodermal appendages [9]. Epithelial stem cells help in the daily normal physiological epidermal renewal and hair follicles regeneration [9]. During wound healing, epithelial stem cells aid in re-epithelialization by differentiating into epithelial cells, mainly keratinocytes and recruiting epidermal progeny cells [9,10]. They also differentiate into glandular and ductal cells to maintain the ducts opening, of sweat and sebaceous glands, to the skin surface. After re-epithelialization, some epithelial stem cells differentiate into neogenic hair follicle which forms the hair shaft [9].

## 3. Matrix Metalloproteinases

Matrix metalloproteinases (MMPs) play an essential role in re-epithelialization [11]. They disrupt hemidesmosome attachments to facilitate release of keratinocytes from the basement membrane and migration into the wound. Keratinocytes produce several MMPs that are needed for migration, including MMP-1, -2, -3, -9 and -10 [11]. Tissue inhibitors of metalloproteinase (TIMPs) block MMP activity and also are expressed during healing [12]. If the expression of TIMPs exceeds that of MMPs in the early phases of wound repair, healing is compromised through reduced keratinocyte migration. For example, mice that over-express TIMP-1 in keratinocytes have reduced keratinocyte migration and decrease the rate of skin wound healing by 4.6-fold [12]. Inhibition of MMP9 activity *in vitro* inhibits keratinocyte migration and deletion of MMP-9 *in vivo* in genetically modified mice reduces the rate of healing [13]. *In vivo* study reported that at day 10, 40% of wound closure and re-epithelialization occurred on mice with MMP-9 deletion while the control group had 100% healed wound [13]. Recently, it has been shown that proline rich protein tyrosine kinase 2 (Pyk2) is up-regulated during wound healing and is needed for keratinocyte migration. Pyk2 is induced by wound healing and simulates PKCδ to increase MMP expression and enhances keratinocyte migration [14]. Pyk2 also increases keratinocyte proliferation that enhances re-epithelialization of wound surface. The increased migration and proliferation significantly enhanced the rate of wound closure with Pyk2 in wildtype mice compared with Pyk2 deficient mice [14].

Too much or prolonged MMP activity is thought to contribute to poor healing seen in diabetic and chronic wounds [11,15]. Chronic and diabetic wounds have increased MMP-1, -2, -8 and -9 and reduced levels of TIMP-1 and -2 [15]. Thus, down regulation of MMPs by TIMPs is important in later stages of healing [8,11]. When MMPs remain high and TIMPs are not sufficiently induced, wounds become chronic. This may be due in part to prolonged inflammation that promotes the expression and activation of MMPs [8,11]. The prolongation of the inflammatory phase is linked to the persistence of bacteria or a significant decrease in removal of debris [6,8]. During prolonged inflammation, neutrophils break down extra-cellular matrix proteins and cause damage to the healthy adjacent tissue, which inhibits keratinocyte migration. Thus, increased MMP activity at later stages damages extracellular matrix and impedes the resolution of inflammation and healing [11].

## 4. Oxidative Stress and Wound Healing

Reactive oxygen species (ROS) are formed by free oxygen radicals and produce oxidative stress [16,17]. Examples of oxygen free radicals are superoxide (O_2_^−^) and hydroxyl radicals (OH^−^), and hydrogen peroxide (H_2_O_2_) [18]. ROS are produced by leukocytes, fibroblasts, keratinocytes and endothelial cells [18]. Low levels of ROS are important in wound repair by protecting the injured area against microbes along with enhancing angiogenesis [19]. Normal ROS levels promote the collagenase activity MMP-1 and the EGF signaling which help wound re-epithelialization through maintaining normal keratinocytes migration and proliferation [20]. In contrast, large amounts of ROS can damage cellular constituents like DNA, lipids, and protein. High levels of ROS also impair cellular functions like cell migration, cell proliferation, and extracellular matrix (ECM) synthesis of fibroblasts and keratinocytes [17]. Normal ROS levels aid in the production of collagen I, III, IV and their subsequent cross linking, and the generation of myofibroblasts. This helps in bringing the wound edges together, which makes the re-epithelialization process faster [20]. High levels of oxidative stress also increase apoptosis of keratinocytes when cultured in a hyperglycemic media, leading to delayed wound healing compared to normoglycemic media [21]. Hyperglycemia therefore increases damage from ROS, which may contribute to poor wound healing in diabetics. High levels of ROS damages fibroblasts, causing them to become senescent and lose the ability to produce extracellular matrix [20]. Senescent fibroblasts also affect wound repair because they are resistant to apoptosis, allowing them to accumulate in the wound area and increase the production of MMPs and pro-inflammatory cytokines [20,22,23]. ROS stimulate apoptosis through the C-Jun *N*-terminal kinase (JNK) pathway and stimulates translocation of JNK to the mitochondria. This causes inhibition of the anti-apoptosis factors like B cell lymphoma-2 (Bcl-2) and activation of the pro-apoptosis factors like Bcl-2-associated X protein (Bax) which impairs wound healing [24].

## 5. Forkhead BoxO-1 (FOXO1) and Re-Epithelialization

Transcription factors are important in coordinating events that are needed for wound healing. C-Jun is a transcription factor that has a role in normal epidermal growth through keratinocytes recruitment and proliferation. A study on wounded mice showed that C-Jun is needed for expression of heparin binding-EGF-like growth factor (HB-EGF), which is produced by monocytes and macrophages to enhance wound healing. Thus, C-Jun deletion leads to a reduction in HB-EGF, which in turn decreases keratinocyte migration and proliferation. A rescue experiment by HB-EGF reversed the negative effect of C-Jun deletion [25]. Peroxisome proliferator activated receptors (PPARs) are transcription factors that are expressed as a result of wounding and enhance keratinocytes migration, proliferation and differentiation [26,27]. In an * in vivo* study, wounded mice with PPARα deletion showed a delay in wound healing by 1–2 days. The delay occurred during the early phase of healing with decreased keratinocyte migration and proliferation [28]. PPARβ deletion showed 2–3 days delay in wound healing because of the decrease in keratinocytes adhesion and migration to the wound area [28].

FOXO1 is a member of the forkhead transcription factors in the O-box sub-family. There are four members, FOXO-1, -3, -4 and -6 [16]. The FOXO transcription factors bind to a highly conserved DNA response element. FOXO1 and FOXO3 are the most closely related, and in some cases have overlapping function while in others they do not [29]. FOXO1 regulates transcription of many different classes of genes depending upon the cell type and nature of the stimulus [29]. FOXO1 has important tumor suppressor functions due to its pro-apoptotic effect through regulation of apoptotic genes. It also plays a role in the immune response by protecting hematopoietic stem cells from oxidative stress [30].

**Figure 1:**
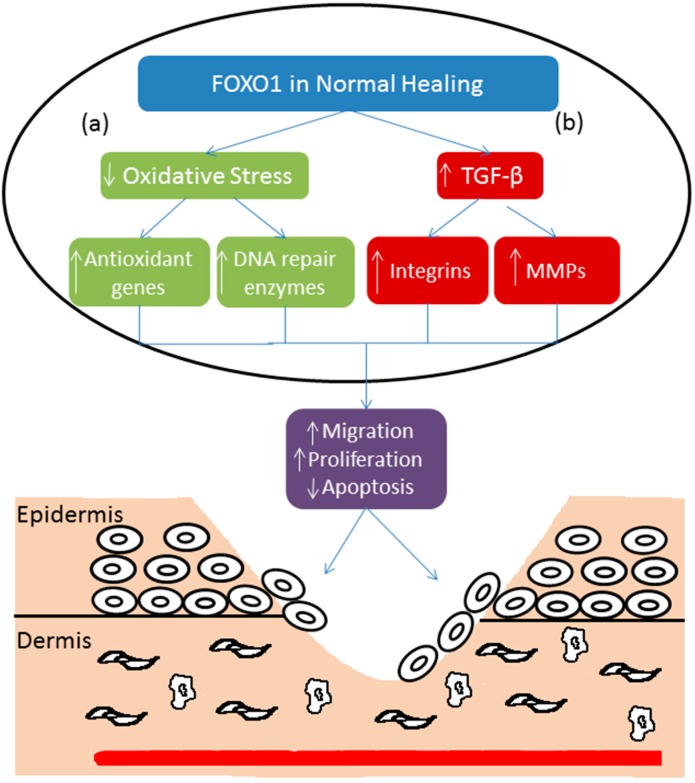
(**a**) Oxidative stress increases in the inflammatory phase of wound healing. FOXO1 down-regulates oxidative stress by activating antioxidant genes and DNA repair enzymes. This facilitates keratinocyte migration and proliferation and decreases keratinocyte apoptosis, which improves wound healing; (**b**) FOXO1 stimulates TGF-β promoter activity resulting in the upregulation of TGF-β expression. Increased TGF-β stimulates expression of integrins and matrix metalloproteinases to improve wound healing through increased keratinocyte migration and decreased apoptosis.

FOXO1 activity is regulated by acetylation, phosphorylation and ubiquitination [16]. After activation, FOXO1 translocates to the nucleus and regulates transcription of other genes [16,31]. In the normal epidermis FOXO1 has a low level of expression and activation, both of which are significantly increased by wounding [31,32]. Wound healing increases the expression of genes with FOXO1 response elements [32]. One of the functions of FOXO1 in wound healing is protecting keratinocytes from oxidative stress by regulating antioxidant genes such as glutathione peroxidase 2 (GPX-2) [33]. FOXO1 also regulates DNA repair enzymes like GADD45, which further protects cells from ROS. FOXO1 deletion in keratinocytes increases oxidative stress by 38% [33]. High level of oxidative stress in turn impairs keratinocyte migration (Figure 1). In support of this, the negative effect of FOXO1 knockdown on keratinocyte migration *in vitro* can be rescued by treatment with an antioxidant [33]. FOXO1 deletion also leads to a 3.7-fold upregulation effect of oxidative stress on apoptosis. When cells are exposed to H_2_O_2_ FOXO1 deletion further increases the level of keratinocyte apoptosis induced by oxidative stress [33].

TGF-β has many cellular functions such as cell cycle regulation [34]. TGF-β binds to type I receptors that induce a signaling cascade that leads to Smad2 and Smad3 phosphorylation and binding to Smad4. The Smad2/3/4 complex translocates to modulate activation or deactivation of many transcription factors [35]. TGF-β facilitates re-epithelialization largely through enhancing migration of keratinocytes after ligand binding + to cell surface receptors [36,37]. It also attracts macrophages and fibroblasts to wound areas to improve healing [37,38]. Integrins are receptors that bind extracellular matrix, which can also induce an intracellular signaling cascade [39]. Integrins thus have a role in re-epithelialization by facilitating migration and proliferation of keratinocytes [40]. Integrin α5, αv, and β5 increase in human keratinocyte treated with TGF-β, which enhance re-epithelialization by increasing the migratory keratinocytes [41].

Keratinocyte migration is an important step in the wound healing process. Migration is dependent on the optimal expression of integrins, MMPs, and factors that stimulate migration such as TGF-β1 [33]. FOXO1 affects keratinocyte migration by regulation of integrins and TGF-β1 [33] (Figure 1). *In vivo*, the deletion of FOXO1 in keratinocytes of mice cause a 50% decrease in migration and 22% decreases in proliferating keratinocytes. FOXO1 deletion *in vivo* decreases the expression of TGF-β1 by 71% compared to the control group and treatment with TGF-β1 rescues the negative effect of FOXO1 on keratinocyte migration [33]. As mentioned before, excessive cell death in the early and middle phases of the wound healing may be detrimental to repair processes. Also *in vivo* deletion of FOXO1 in mice keratinocytes causes increased apoptosis by 1.6-fold via reduced expression of TGF-β. FOXO1 deletion increases pro-apoptotic genes like *Bim*, which lead to loss of keratinocytes. Therefore, FOXO1 is required to prevent increased apoptosis under normal conditions [33]. In contrast FOXO3 knockout mice were reported to have significantly accelerated rate of wound healing [42]. We have found that FOXO3 did not affect wound healing behavior of keratinocytes *in vitro* [33], which agrees well with reports that FOXO3 deletion does not affect wound healing *in vivo* [43]. In contrast to lineage specific deletion of FOXO1, non-specific FOXO1 reduction in all cell types was examined using heterozygous *Foxo1*^+/−^ mice [43]. These mice exhibited accelerated skin healing that was linked to an attenuated inflammatory response and a reduced expression of growth factors. This is likely to be due to the broad effects of FOXO1 deletion on multiple cell types since we have shown that keratinocyte specific deletion of FOXO1 impairs healing [33]. For example we have shown that FOXO1 deletion impairs the activity of dendritic cells, which causes a compensatory increase in inflammation [44] (Xiao, *et al.*, unpublished data). Alternatively it could represent the difference between heterozygous and homozygous FOXO1 deletion.

## 6. FOXO1 and Diabetic Wound Healing

Ak strain transforming (Akt), also called protein kinase B (PKB), is kinase that regulates apoptosis, migration, proliferation, and other cellular activities. Akt is downstream of insulin signaling and is activated by phosphatidylinositide 3-kinases (PI3K) through the phosphorylation of insulin receptor substrates-1 and -2, which result in Akt activation [16,45]. One of the substrates of Akt is FOXO1, which is inactivated by phosphorylation by Akt [16]. In addition, Akt has other downstream targets such as BAD, MDM2, glycogen synthase kinase 3 (GSK3), and p27 [46]. In general, there are three mammalian Akt isoforms (Akt1, Akt2, and Akt3) [47]. In the liver, FOXO1 controls gene expression that promotes gluconeogenesis resulting in high glucose production [16]. In diabetic conditions, insulin resistance causes Akt inactivation leading to greater FOXO1 activation due to reduced Akt activity; FOXO1, in turn, up-regulates genes that promote gluconeogenesis, such as Pgc1α and Atp5b, and increases serum glucose levels [16,48]. In other tissues, such as endothelial cells insulin–PI3K–Akt signaling also leads to deactivation of FOXO1 by increasing FOXO1 transport out of the nucleus [49]. In endothelial cells and pericytes increased FOXO1 activation by inflammatory mediators or advanced glycation end products, both of which are elevated in diabetes, leads to greater cell death and may be an important component of some microvascular complications such as diabetic retinopathy [50,51]. Microvascular complications may contribute to impaired wound healing by affecting the function of vascular cells and surrounding basement membrane [52].

Diabetic wounds have decreased cellular migration and proliferation, and elevated levels of apoptosis [53,54]. Keratinocytes at the wound edge in diabetic patients exhibit reduced migration by 60% [44,54]; Fibroblasts and keratinocytes from diabetic individuals or in high glucose have decreased migration, proliferation, and increased apoptosis [55,56,57]. High levels of glucose appear to be a factor in diabetes as it impaired fibroblasts proliferation around 40% and also decreased fibroblasts migration by 60% [56,58]. Wound closure is important in preventing infection, and delayed closure in diabetics increases the likelihood of infection [1]. Furthermore, bacteria have direct effects on keratinocytes and can further impede keratinocyte proliferation and migration and enhance keratinocyte cell death [59]. Resistance to bacterial infection in the mouth may be particularly important since mice with diminished host response (*IL-1*^−/−^) have impaired healing of excisional wounds in the oral cavity while the same mice have normal dermal healing [60]. Part of the effect of bacteria on kerationcytes is mediated by FOXO1 and FOXO3, which regulate expression of genes associated apoptosis (BID and TRADD), inflammation (TLR-2 and -4) and barrier function (integrin β-1, -3 and -6) [61]. In addition bacteria can indirectly limit repair through increased inflammation [62].

**Figure 2:**
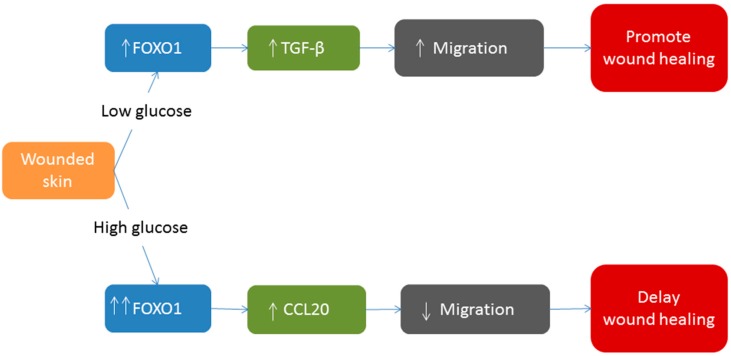
In low glucose conditions, FOXO1 promotes wound healing by increasing TGF-β levels. This leads to an increase in keratinocyte migration and re-epithelialization. However, in high glucose conditions FOXO1 does not stimulate an increase in TGFβ1. Instead, FOXO1 increases inflammatory gene expression (increased CCL20), which interferes with keratinocyte migration. Thus, in standard glucose levels FOXO1 promotes healing but in high glucose FOXO1 is detrimental to healing by a switch in the genes that it regulates (see [44]).

Myofibroblasts are specialized fibroblasts that produce extracellular matrix and exert contractile forces to contract the wound. TGF-β signaling appears to be important in stimulating myofibroblasts. Reduced TGF-β may contribute to diabetes impaired wound contraction [63]. Diabetic wounds have increased levels of TNF-α [64]. *In vivo*, inhibiting TNF-α increases fibroblast proliferation more than 2-fold in diabetic wounds but it has little effect in wounds of normoglycemic mice [57]. Elevated TNF-α in diabetic wounds also increases fibroblast apoptosis about 5-fold [57,65] and apoptosis is 20% higher in skin biopsies from diabetic foot ulcers than control group [66,67]. Increased levels of TNF-α in diabetic wounds also increase the expression and activation of FOXO1 [55,57]. While FOXO1 is crucial for normal wound healing, its hyperactivation in diabetic wounds is thought to be problematic [44]. Elevated activation of FOXO1 increases apoptosis [30] and is associated with increased inflammation [53] that may lead to poor wound healing in hyperglycemic conditions. Under normal wound healing conditions FOXO1 enhances migration of keratinocytes. Paradoxically, hyperactivation of FOXO1 in high glucose conditions *in vitro* or in diabetic mice *in vivo* decreases keratinocyte migration by more than 50% [44]. Mechanistically, FOXO1 impairs keratinocyte migration in high glucose through the production of pro-inflammatory factors [44]. Although FOXO1 is not typically thought of as a pro-inflammatory transcription factor it has been shown to contribute to expression of inflammatory mediators in a number of cell types including dendritic cells, keratinocytes, chondrocytes and macrophages [53,62,68,69]. In addition, FOXO1 does not upregulate TGF-β1 expression in high glucose, whereas in standard glucose conditions FOXO1 plays a positive role by inducing TGF-β1 transcription. This represents another paradox, as diabetes leads to greater activation of FOXO1 but this greater activation is associated with a reduced ability to stimulate TGF-β1 transcription [44]. In addition, the hyperactivation of FOXO1 in diabetic wounds leads to increased expression of pro-apoptotic genes, which is consistent with reports that FOXO1 mediates a 50%–70% upregulation of proapoptotic genes during fracture healing [70]. This is in contrast to normal healing in which FOXO1 is needed to prevent apoptosis. Figure 2 shows the differences of FOXO1 role on wound healing in high glucose condition and in low glucose condition. Therefore, it appears that diabetes alters the function of FOXO1 so that it switches from being a pro-healing anti-apoptotic transcription factor to one that fails to up-regulate TGF-β1 and becomes pro-inflammatory and pro-apoptotic.

## 7. Conclusions

Wound healing is a complex process which depends on many factors for its success. Migration, proliferation, and apoptosis are important events in wound healing. Recent studies have shown that FOXO1 is an important regulator of wound healing and that diabetes influences the effect of FOXO1. In normal wound healing, FOXO1 plays a positive role by inducing TGF-β expression, which is needed for keratinocyte migration. In addition FOXO1 has an anti-oxidant role that protects keratinocytes against ROS. In high glucose conditions FOXO1 has the opposite effect and fails to stimulate TGF-β. Thus, in diabetic animals FOXO1 fails to induce a positive factor, TGF-β but instead stimulates expression of factors that interfere with keratinocyte migration to delay healing.
